# Perioperative Analgesia for Foot and Ankle Surgery: A Comprehensive Review

**DOI:** 10.3390/jcm14176301

**Published:** 2025-09-06

**Authors:** Giuseppe Pascarella, Eugenio De Quattro, Alessandro Strumia, Romualdo Del Buono, Francesca Gargano, Alessandro Ruggiero, Sabrina Migliorelli, Lorenzo Schiavoni, Alessia Mattei, Rita Cataldo, Massimiliano Carassiti, Felice Eugenio Agrò, Fabio Costa

**Affiliations:** 1 Unit of Anesthesia and Intensive Care, Fondazione Policlinico Universitario Campus Bio-Medico, 00128 Rome, Italye.dequattro@policlinicocampus.it (E.D.Q.); sabrina.migliorelli@policlinicocampus.it (S.M.); r.cataldo@policlinicocampus.it (R.C.);; 2 Unit of Anesthesia, Resuscitation, Intensive Care and Pain Management, ASST Gaetano Pini, 20122 Milano, Italy; 3 Research Unit of Anesthesia and Intensive Care, Department of Medicine, Università Campus Bio-Medico di Roma, Via Alvaro del Portillo, 21, 00128 Roma, Italy

**Keywords:** pain, ankle surgery, regional anesthesia, pain management, early mobilization, multimodal analgesia, ambulatory surgery, opioid-sparing

## Abstract

Foot and ankle surgery is often associated with significant postoperative pain that may delay mobilization and recovery. For this reason, effective perioperative analgesia is essential to improve outcomes, minimize opioid use, and enable safe early discharge when appropriate. This review provides an updated overview of regional anesthesia techniques for foot and ankle surgery, highlighting anatomical considerations, ultrasound guidance, and patient-tailored block selection. Different techniques will be specifically addressed over neuraxial and nerve blocks, such as the Mayo block and intravenous regional anesthesia (Bier block), as well as the emerging WALANT approach for selected cases. Ultrasound guidance has become a gold-standard, enhancing precision and safety compared to landmark-based methods. Multimodal analgesia combining regional blocks with non-opioid medications and adjuvants like intravenous dexamethasone further optimizes pain control while limiting opioid requirements. In ambulatory settings, motor-sparing techniques and short-acting spinal agents are emphasized to support rapid recovery and same-day discharge. By integrating anatomical knowledge with ultrasound and multimodal strategies, perioperative pain management for foot and ankle surgery can be tailored to balance effective analgesia with early mobilization and patient safety.

## 1. Introduction

Foot and ankle surgery encompasses a broad spectrum of elective and trauma procedures, including hallux valgus correction, hammertoe repair, metatarsal osteotomies, Achilles tendon repairs, and fracture fixation. Postoperative pain is a significant concern, with up to 60% of patients experiencing moderate to severe pain, potentially leading to delayed mobilization, prolonged recovery, and increased opioid consumption [1]. In this context, regional anesthesia has emerged as a cornerstone of perioperative pain management. It offers effective intraoperative anesthesia and extended postoperative analgesia, while minimizing systemic opioid-related side effects [2]. Compared to general anesthesia, regional techniques can provide targeted analgesia with a lower risk of complications and improved patient tolerance [3]. However, the choice of the optimal block should depend on multiple factors, including type of surgery, hospital admission (in or out-patient), human factors, and patient characteristics [4]. This narrative review aims to provide a comprehensive overview of regional anesthesia techniques for foot and ankle surgery, correlating anatomical knowledge with clinical application to optimize perioperative pain management. Particular emphasis will be placed on ultrasound-guided techniques, which have significantly improved the safety, precision, and efficacy of nerve blocks during the last decades [5,6].

## 2. Methods

A comprehensive literature search was conducted in PubMed/MEDLINE (https://pubmed.ncbi.nlm.nih.gov, accessed on 26 July 2025), Embase (https://www.embase.com, accessed on 26 July 2025), and the Cochrane Library (https://www.cochranelibrary.com, accessed on 26 July 2025) to identify studies focused on regional and multimodal analgesia for foot and ankle surgery. The PubMed search strategy included a combination of MeSH terms and free-text keywords:

(“Foot Surgery”[MeSH] OR “Ankle Surgery”[MeSH] OR “Foot Injuries/surgery”[MeSH] OR “Ankle Injuries/surgery”[MeSH] OR “foot surgery” OR “ankle surgery” OR “foot and ankle surgery” OR “foot and ankle procedures”)

AND (“Regional Anesthesia”[MeSH] OR “Anesthesia, Conduction”[MeSH] OR “Nerve Block”[MeSH] OR “Peripheral Nerve Block” OR “Popliteal Block” OR “Ankle Block” OR “sciatic nerve block” OR “femoral nerve block” OR “regional block” OR “conduction anesthesia” OR “Mayo Block” OR “Bier Block” OR “Intravenous Regional Anesthesia” OR “WALANT” OR “Wide Awake Local Anesthesia No Tourniquet”)

AND (“Multimodal Analgesia”[MeSH] OR “Pain Management”[MeSH] OR “Postoperative Pain/prevention and control”[MeSH] OR “analgesia” OR “multimodal analgesia” OR “opioid-sparing” OR “non-opioid analgesics” OR “perioperative pain management”).

Search strategies were adapted for each database using the appropriate syntax and controlled vocabulary (e.g., Emtree for Embase). All identified studies were screened for quality and clinical relevance by two independent reviewers (FC and GP).

## 3. Innervation of the Foot and Ankle

The sensory and motor innervation of the foot and ankle is primarily derived from the sciatic and saphenous nerves, branches of the lumbosacral plexus. The sciatic nerve (roots L4-S3) descends through the posterior thigh and bifurcates in the popliteal fossa into the tibial and common peroneal nerves [7]. The tibial nerve descends and passes posterior to the medial malleolus before branching into medial and lateral plantar nerves. The plantar nerves provide sensory innervation to the sole of the foot, and the intrinsic muscles of the foot (e.g., flexor digitorum brevis and abductor hallucis). Specifically, the medial plantar nerve innervates the medial two-thirds of the sole of the foot, and the lateral plantar nerve innervates the lateral third and the small muscles of the foot [2]. The common peroneal nerve wraps around the fibular neck after branching from the sciatic nerve and then derives into the deep peroneal nerve (DPN) and superficial peroneal nerve (SPN). DPN is responsible for motor innervation to the muscles of the anterior compartment of the leg (e.g., tibialis anterior) and sensory innervation to the interdigital space between the first and second toes. SPN provides sensation to the dorsum of the foot and the lateral part of the lower leg and provides motor innervation to the peroneus longus and brevis muscles. The medial side of the foot and ankle are also innervated by the saphenous nerve (sensory branch of the femoral nerve), which is the longest branch of the femoral nerve running along the great saphenous vein. It provides cutaneous sensation to the medial leg, ankle and foot. A comprehensive understanding of this intricate anatomy is essential for effective regional anesthesia, as it allows precise targeting of the nerves responsible for sensory and motor function in the surgical area, optimizing perioperative pain management.

**Figure 1:**
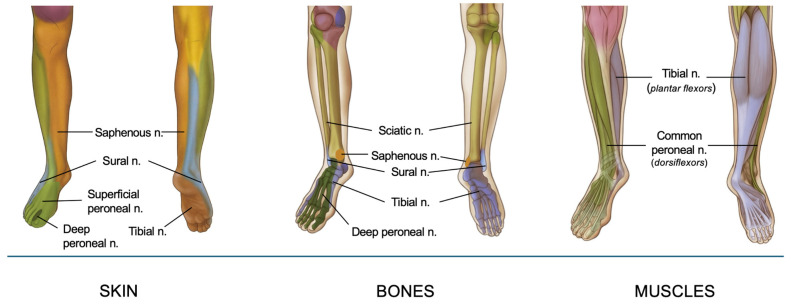
Innervation of foot and ankle.

Innervation of foot and ankle is resumed in Figure 1.

A synthesis of foot and ankle innervation, organized by cutaneous, muscular, and bony structures.

## 4. Regional Anesthesia Techniques

The administration of anesthesia for foot surgery requires a tailored approach due to the distinct anatomical and functional demands of this region. The use of regional anesthesia in such surgical interventions offers several advantages, including reduced pain scores, decreased opioid consumption, and enhanced patient outcomes. Several studies have suggested that patients receiving regional anesthesia for foot and ankle surgeries reported progressively lower pain scores with low narcotic use up to 5–6 days postoperatively [2]. Moreover, the integration of ultrasound guidance has been instrumental in revolutionizing these techniques, enhancing precision, reducing complications, and improving patient outcomes. This section explores the indications, procedural details, and evidence-based benefits of these techniques in the context of foot surgery.

### 4.1. Spinal Anesthesia

Spinal anesthesia remains a reliable and effective technique for foot and ankle surgery, particularly when a thigh tourniquet is required [8]. In such cases, it provides dense intraoperative analgesia and effective tourniquet tolerance, avoiding deep sedation or the use of proximal peripheral nerve blocks—such as femoral or high sciatic blocks—which may lead to prolonged postoperative motor block. The choice of local anesthetic agent for spinal anesthesia should be tailored to the expected duration of surgery. Agents with a shorter duration of action (i.e., prilocaine or 2-chloroprocaine) are preferred for brief procedures to minimize motor block and enable rapid recovery, as demonstrated in day-case settings. For longer surgical interventions, longer-acting agents such as bupivacaine, levobupivacaine, or ropivacaine are typically chosen due to their prolonged duration of anesthesia.

This decision should involve a shared discussion between the anesthesiologist, the surgeon, and the patient, balancing surgical needs, patient comorbidities, and recovery goals.

Although spinal anesthesia offers excellent intraoperative conditions, its analgesic effect typically does not extend into the postoperative period. For this reason, it is often integrated with peripheral nerve blocks targeting the distal branches of the sciatic nerve, in order to prolong analgesia and reduce opioid consumption after surgery. Nonetheless, spinal anesthesia is associated with potential adverse effects, including hypotension, urinary retention, bradycardia, and post-dural puncture headache [9].

### 4.2. Popliteal Sciatic Nerve Block

The popliteal sciatic nerve block (PSNB) is one of the most frequently used techniques for foot and ankle surgery, including Achilles tendon repair, ankle fracture fixation, and forefoot reconstruction [7]. The block is performed in the popliteal fossa before the sciatic nerve divides into the tibial and common peroneal nerves, enabling effective anesthesia of the distal lower limb. Historically, several landmark-based techniques have been developed using peripheral nerve stimulation (PNS) to identify the nerve structures. These remain useful when ultrasound is unavailable or as an adjunct to enhance accuracy. Two main approaches were commonly used: the posterior approach, with the patient in prone decubitus position, the needle was inserted between the tendons of the hamstring muscles (semitendinosus/semimembranosus and biceps femori) 7–10 cm cranial to the popliteal crease; the lateral approach, with the patient in supine position, the needle was inserted in the groove between the vastus lateralis and biceps femoris, approximately 10 cm proximal to the lateral femoral condyle, it is advanced until contact with the femur, then withdrawn to the level of the skin and redirected approximately 30° posteriorly [10].

We highlight that lateral positioning is generally preferred to the prone position due to improved patient comfort.

**Figure 2:**
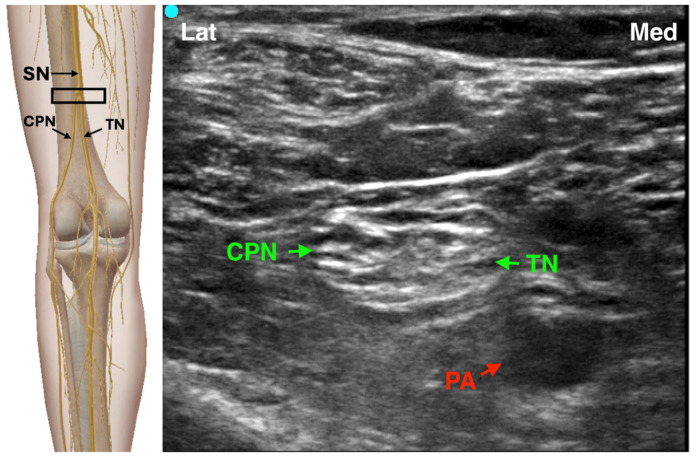
Ultrasound-guided Popliteal Sciatic nerve Block SN: Sciatic Nerve; TN: Tibial Nerve; CPN: Common Peroneal Nerve; PA: Popliteal Artery; The black rectangle indicates the placement of the ultrasound probe on the anatomical area.

During PNS-guided blocks, stimulation is typically started at 1–2 mA and reduced to 0.5 mA once a motor response is elicited. Plantarflexion and inversion suggest tibial nerve component stimulation, while dorsiflexion and eversion indicate DPN component stimulation. The introduction of ultrasound guidance has transformed PSNB practice, offering real-time visualization of the sciatic nerve and surrounding anatomy, and improving safety and block success [11,12,13]. The procedure can be performed with the patient in lateral or prone position using a high-frequency linear probe. The sciatic nerve is identified in the popliteal fossa, and the needle is advanced in- or out-of-plane to deposit 20–30 mL of local anesthetic (e.g., ropivacaine 0.5% or bupivacaine 0.25%) around the nerve [13] (Figure 2). Recent developments include the sub-sheath and extra-sheath approaches, where local anesthetic is injected within or outside the paraneural sheath, respectively. Sub-sheath injections may yield faster onset, although with a higher risk of nerve injury [14,15]. In order to avoid such events, the adjunct of opening injection pressure monitoring may be helpful to ensure a more precise and safe needle positioning, though further studies are warranted to assess long-term outcomes and optimal dosing strategies [16]. Clinical studies show that PSNB provides prolonged postoperative analgesia, reduces opioid requirements, and avoids systemic side effects typical of neuraxial anesthesia. Continuous popliteal sciatic nerve block plays a relevant role in managing more complex and painful procedures, showing comparable outcomes in terms of postoperative pain relief and patient satisfaction. However, current evidence remains limited regarding its safety profile, and further large-scale studies are required to validate its long-term efficacy and safety [17]. Compared to spinal anesthesia, it has demonstrated superior patient satisfaction and fewer complications such as hypotension or urinary retention, making it an attractive option for hallux valgus and other forefoot surgeries [18].

### 4.3. Ankle Block

The ankle block is a form of regional anesthesia that targets the five terminal nerves of the foot: the tibial, deep peroneal, superficial peroneal, saphenous, and sural nerves. It is commonly used for forefoot procedures such as bunionectomy, toe amputation, or excision of Morton’s neuroma [19,20]. However, a high-ankle block (10–15 cm proximally to the malleolus plane) has been showed effective also for ankle surgery [21]. Each nerve must be individually identified and anesthetized to achieve complete sensory blockade of the foot. Ankle blocks have a rapid onset (typically within a few minutes) and cause minimal motor block, mainly limited to the plantar interosseous muscles. The block offers high selectivity, as it does not involve the leg or thigh and can therefore be performed only when a tourniquet is applied at the ankle.

Patients can usually ambulate after surgery, although caution is advised due to reduced sensation. Ultrasound guidance improves the accuracy and safety of ankle blocks by enabling direct visualization of neural structures. Abrahams et al. demonstrated that ultrasound increases success rates, reduces procedure time, and lowers the risk of complications compared to nerve stimulation alone; moreover, these distal techniques mainly involve sensitive nerves and motor response to PNS is not reliable [22]. Similarly, Olofsson et al. found that for forefoot surgery, a sciatic nerve block did not significantly prolong analgesia compared to an ankle block, with comparable opioid requirements but with consequent motor impairment [23]. Not by chance, the latest PROSPECT guidelines for hallux valgus repair recommend the ankle block as the preferred regional technique, as it avoids the need for general or spinal anesthesia and supports faster patient ambulation [24]. Below, we provide a detailed explanation of all the nerve blocks to be targeted when performing an ankle block.

#### 4.3.1. Tibial Nerve Block

**Figure 3:**
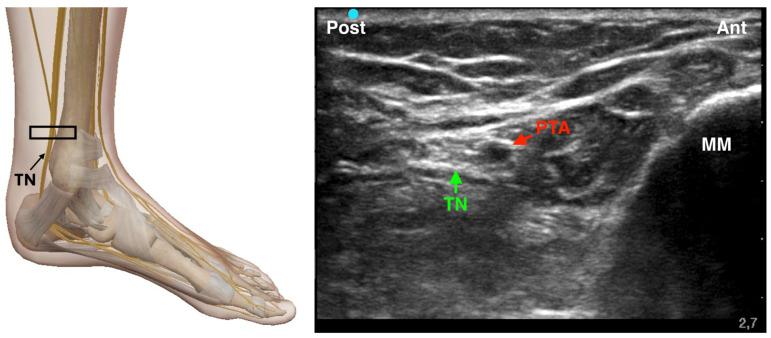
Ultrasound-guided Tibial Nerve Block. TN: Tibial Nerve; PTA: Posterior Tibial Artery; MM: Medial Malleolus; The black rectangle indicates the placement of the ultrasound probe on the anatomical area.

The tibial nerve is the largest branch of the sciatic nerve and provides sensory innervation to the plantar surface of the foot, as well as motor control to its intrinsic muscles. It is located posterior to the medial malleolus, coursing alongside the posterior tibial artery and veins within the tarsal tunnel. Tibial nerve blockade can be performed using either a landmark-based or an ultrasound-guided approach. The traditional technique, described by Brown, relies on palpation of anatomical landmarks—namely, the Achilles tendon and the medial malleolus—rather than direct nerve visualization [20]. In this method, the needle is inserted medial to the Achilles tendon at the superior border of the medial malleolus and directed toward the malleolus. If paresthesia is elicited in the foot, local anesthetic is injected; if not, the needle is repositioned by advancing, altering its angle, or contacting bone and withdrawing slightly (2–3 mm) before injection. In contrast, the ultrasound-guided technique offers direct visualization of the tibial nerve and surrounding structures, enhancing both precision and safety [25]. A high-frequency linear transducer is placed in a transverse orientation posterior to the medial malleolus. The tibial nerve appears as a hyperechoic structure adjacent to the posterior tibial artery (Figure 3). Using an in-plane technique, a 22-gauge needle is advanced from posterior to anterior, and 3–5 mL of local anesthetic (e.g., 0.5% ropivacaine or 2% mepivacaine) is injected circumferentially around the nerve. Aspiration prior to injection is essential to avoid intravascular administration. Both techniques can be effective; however, Redborg et al. demonstrated that ultrasound guidance—particularly through identification of the posterior tibial artery—yields a higher success rate compared to landmark-based methods, which depend more heavily on surface anatomy and require smaller volumes of local anesthetic [6].

#### 4.3.2. Deep Peroneal Nerve Block

**Figure 4:**
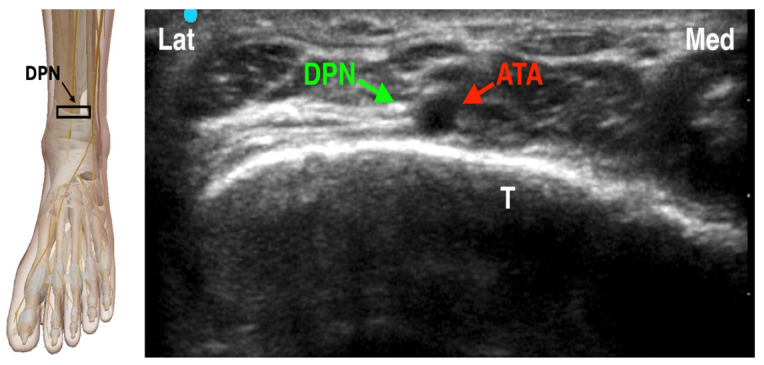
Ultrasound-guided Deep peroneal Nerve Block. DPN: Deep Peroneal Nerve; ATA: Anterior Tibial Artery; T: Tibia. The black rectangle indicates the placement of the ultrasound probe on the anatomical area.

The deep peroneal nerve provides motor innervation to the muscles of the anterior compartment of the leg and the extensor digitorum brevis, as well as sensory innervation to the first interdigital space between the hallux and second toe. It descends along the anterior tibial artery, medial to the interosseous membrane and deep to the extensor retinaculum at the level of the ankle [26]. When performing an ultrasound-guided deep peroneal nerve block at the ankle, the nerve typically appears lateral or anterior to the anterior tibial artery (ATA), just superficial to the hyperechoic cortex of the distal tibia. Key anatomical landmarks include the tibialis anterior tendon medially and the extensor digitorum longus and extensor hallucis longus tendons laterally. Dynamic scanning facilitates identification by highlighting the nerve’s relative movement to adjacent structures and its characteristic speckled echotexture. In clinical practice, the ultrasound probe is placed transversely on the dorsum of the foot just proximal to the ankle joint. The DPN appears as a small hyperechoic structure adjacent to the ATA (Figure 4). Using an in-plane approach, a 22- to 25-gauge needle is advanced, and 2–3 mL of local anesthetic is deposited around the nerve to achieve effective blockade [19,27,28].

#### 4.3.3. Superficial Peroneal Nerve Block

**Figure 5:**
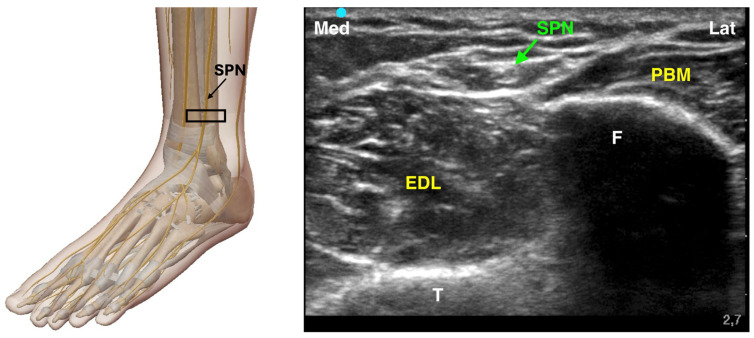
Ultrasound-guided Superficial Peroneal Nerve Block. SPN: Superficial Peroneal Nerve; F: Fibula; T: Tibia; EDL: Extensor Digitorum Longus Muscle; PBM: Peroneus Brevis Muscle; The black rectangle indicates the placement of the ultrasound probe on the anatomical area.

The superficial peroneal nerve provides sensory innervation to most of the dorsum of the foot. It originates from the common peroneal nerve and runs subcutaneously in the distal third of the leg. Due to its highly variable branching pattern, ultrasound guidance is recommended to increase the accuracy of the block. The SPN travels deep to the muscular fascia, positioned between the extensor digitorum longus (EDL) anteriorly and the fibularis longus (FL) and fibularis brevis (FB) posteriorly. As it descends, the SPN pierces the muscular fascia to divide into its terminal branches, which supply sensory innervation to the dorsal aspect of the foot. To perform an ultrasound-guided SPN block, the probe is typically placed proximal to the lateral malleolus and scanned cephalad along the fibula with the patient in a supine position. The nerve can be identified as it crosses the muscular fascia, distinguishing the adjacent muscle groups (Figure 5). Dynamic scanning helps to trace the nerve’s course and confirm its location. A 25-gauge needle is then advanced, and 3–5 mL of local anesthetic is injected either superficial or deep to the muscular fascia. Since the SPN may branch before fully emerging through the fascia, the block should be performed at an appropriate level to ensure an effective sensory blockade of the foot [19,27].

#### 4.3.4. Saphenous Nerve Block

**Figure 6:**
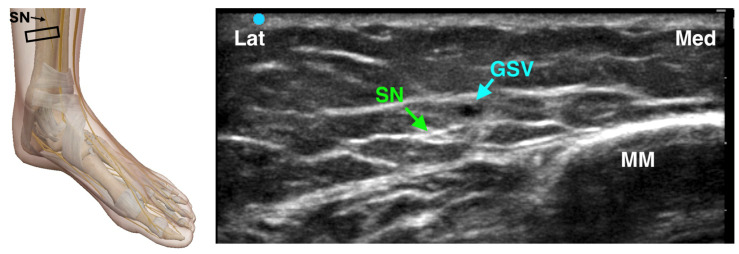
Ultrasound-guided Saphenous Nerve Block. SN: Saphenous Nerve; GSV: Great Saphenous Nerve; MM: Medial Malleolus; The black rectangle indicates the placement of the ultrasound probe on the anatomical area.

The saphenous nerve is a purely sensory branch of the femoral nerve that provides innervation to the medial aspect of the ankle and foot. It runs in close proximity to the great saphenous vein, offering a useful vascular landmark for nerve localization. In the landmark-based technique, a semicircular subcutaneous injection of 3–5 mL of local anesthetic is administered approximately 2 cm above the medial malleolus, on either side of the great saphenous vein, which serves as the primary reference point. For an ultrasound-guided approach, the probe is positioned anterior to the medial malleolus in a transverse plane to visualize the nerve adjacent to the vein (Figure 6). The saphenous nerve may appear either superficial or deep to the great saphenous vein, and a perivascular injection is used to ensure effective anesthesia. Given its superficial location, a field block technique is often appropriate. The need for a saphenous nerve block depends on the planned surgical procedure: it is particularly relevant for proximal osteotomies but is generally unnecessary for more distal foot surgeries [29].

#### 4.3.5. Sural Nerve Block

**Figure 7:**
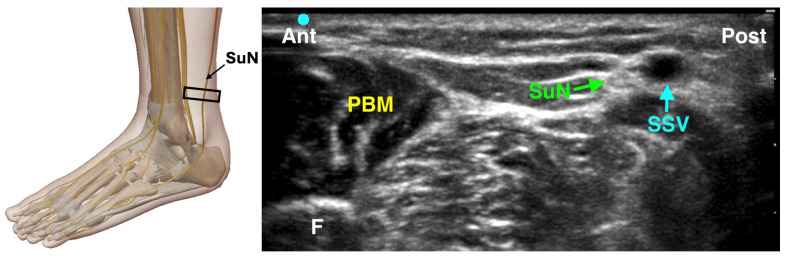
Ultrasound-guided Sural Nerve Block. SuN: Sural Nerve; SSV: Small Saphenous Nerve; PBM: Peroneus Brevis Muscle; F: Fibula; The black rectangle indicates the placement of the ultrasound probe on the anatomical area.

The sural nerve provides sensory innervation to the lateral aspect of the foot, including the lateral malleolus and fifth toe. It typically runs posterior to the lateral malleolus, adjacent to the small saphenous vein [30,31]. The landmark-based technique involves a semicircular subcutaneous injection of 3–5 mL of local anesthetic, 2–4 cm above and behind the lateral malleolus, from the fibula to the Achilles tendon. Although the nerve is very superficial (about 0.5 cm deep), ultrasound guidance offers greater reliability than landmarks alone. For the ultrasound-guided block, the patient is positioned laterally or with the leg internally rotated. The probe is placed transversely just posterior to the lateral malleolus to identify the nerve, which is usually lateral to the small saphenous vein (Figure 7). Distally, the nerve can be seen ‘rolling’ off the Achilles tendon into a boat-shaped space behind the fibula [32]. Using an in-plane technique, a 22–25 gauge needle is advanced anterior-to-posterior and 1–3 mL of local anesthetic is injected around the nerve. If the nerve is not clearly visible, a perivenous injection near the small saphenous vein may still be effective. Due to its variable distribution—sometimes extending to the third toe or medial heel—the sural nerve block may not be indicated for surgeries involving the first and second toes but can improve comfort for lateral foot or ankle procedures.

## 5. Intravenous Regional Anesthesia (Bier Block)

**Figure 8:**
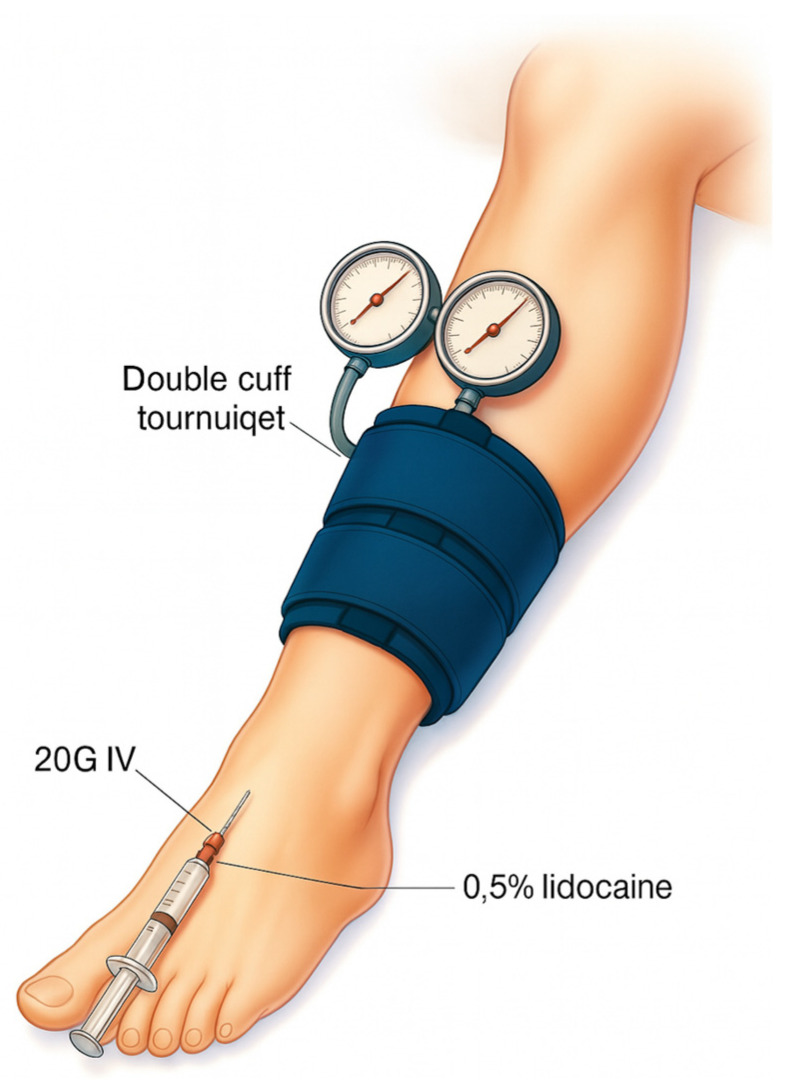
Intravenous Regional Anesthesia (Bier Block) A 20 G intravenous cannula is inserted in the dorsum of the foot. The foot is then exsanguinated using an Esmarch’s bandage and elevating the limb for 3 min. The tourniquet is positioned as in standard procedures, with the distal cuff placed at the level of a single-cuff tourniquet. The proximal cuff is inflated 100 mmHg above the systolic arterial blood pressure, after which the Esmarch bandage is removed. Subsequently 40–50 mL of lidocaine 0.5% are injected through the i.v. cannula, which is then removed. Approximately 25 to 30 min after anesthesia begins, or if the patient reports tourniquet-related pain, the distal cuff is inflated while the proximal cuff is deflated to help reduce discomfort caused by the tourniquet.

Intravenous Regional Anesthesia (IVRA), also known as Bier’s block, is a relatively safe, simple and affordable option for procedures on the upper and lower limbs, originally described by August Bier in 1908. The procedure itself involves exsanguination of the limb by elevation and Esmarch dressing, followed by inflation of a double tourniquet to prevent systemic absorption of the local anesthetic. A dilute local anesthetic, usually 40–50 mL of lidocaine 0.5% lidocaine, is administered by intravenous infusion [33] (Figure 8). Its use is always limited by tourniquet tolerance and surgical duration, as the tourniquet must be inflated for 20 min to reduce the risk of systemic toxicity from the local anesthetic [34]. However, IVRA remains a well-used technique in emergency departments, outpatient settings, and for higher-risk patients who do not want general anesthesia. This approach can be used to perform short procedures (up to 1 h) such as mass removal, digital nerve reconstruction, surgery for phalangeal fractures or dislocations, and excision of an accessory navicular bone. It offers quick onset/offset, good muscle relaxation and an improved safety profile, especially with modern monitoring and resuscitation equipment. IVRA can also help in some cases of complex regional pain syndrome. The local anesthetics used for IVRA include prilocaine and lidocaine, with several adjuvants being tested for some benefit in analgesia and reduction in post-tourniquet pain. In 2011, Flamer et al. [35] showed in an observational study that ropivacaine is effective for IVRA and provides better quality postoperative analgesia. Although lidocaine and prilocaine are effective, they do not have the added benefit of long-lasting pain relief. This limitation is primarily due to the pharmacokinetics of intravenous regional anesthesia: once the tourniquet is released, systemic circulation resumes, leading to a rapid washout of the local anesthetic from the targeted tissues, thereby abruptly terminating its analgesic effect [35]. Although significant complications of IVRA are rare, there is a risk of systemic toxicity if local anesthetics are not controlled and inadvertently enter the circulation [34].

## 6. Mayo Block

**Figure 9:**
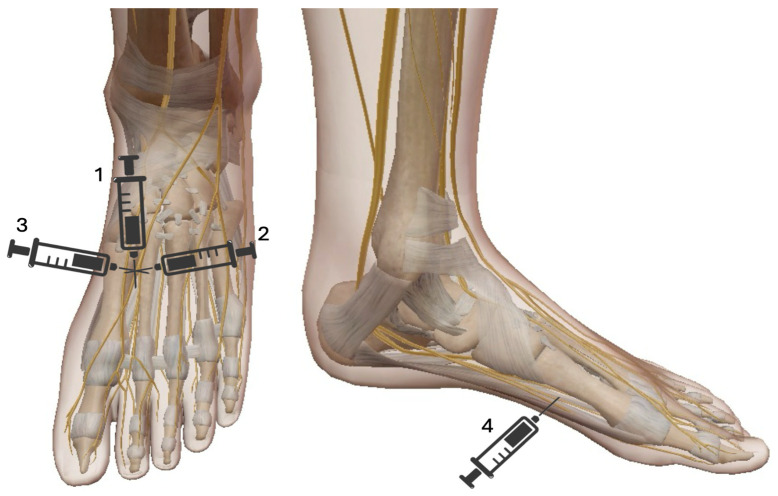
Mayo Block. The Mayo Block consists of 4 different injections each with 3–5 mL of local anesthetic (LA): 1. Insertion point proximally and dorsally within the first intermetatarsal space, followed by advancing the needle in a plantar direction while administering (LA); 2. The needle is then partially withdrawn and redirected medially to create a subcutaneous wheal along this path with an additional injection of LA; 3. the needle is reinserted laterally to raise another subcutaneous wheal with LA along its trajectory; 4. The needle is withdrawn again and advanced plantar-medially beneath the metatarsal bone to deliver of LA from the medial to the lateral aspect.

The Mayo block is a regional anesthetic technique commonly used for hallux valgus surgery and first metatarsal procedures. It provides effective analgesia by blocking the terminal branches of the saphenous, deep peroneal, superficial peroneal, sural, and medial plantar nerves. The Mayo block is suitable for various surgical interventions, including bunionectomy, correction of hallux valgus and hallux rigidus, surgery for ingrown toenails, first metatarsal osteotomies, and other soft tissue procedures involving the great toe and medial foot. [36] This technique involves subcutaneous injection of a local anesthetic (typically lidocaine or bupivacaine) along a horseshoe-shaped line at the base of the first metatarsal (Figure 9). The required volume generally ranges from 10 to 15 mL, depending on patient anatomy and the desired duration of anesthesia. Careful aspiration before injection is essential to avoid inadvertent intravascular administration. The advantages of the Mayo block include effective perioperative and postoperative analgesia, a reduced need for general anesthesia or systemic opioids, facilitation of ambulatory surgery, and a lower risk of systemic side effects compared to general anesthesia. Although considered relatively safe, potential complications include hematoma formation due to accidental vascular puncture, transient nerve injury with paresthesia or dysesthesia, and local anesthetic systemic toxicity (LAST) if excessive doses or intravascular injection occur. Compared to direct infiltration, it requires less local anesthetic, respects tissue planes, and is associated with a very low failure rate, well below 1%. Its use can improve operating room efficiency and reduce the risks associated with general or major conduction anesthesia.

## 7. WALANT

Wide Awake Local Anesthesia No Tourniquet (WALANT) is a field block technique that combines local infiltration of lidocaine with epinephrine (typically at a concentration of 1:100,000) to provide surgical anesthesia and hemostasis without the need for sedation or tourniquet application. Originally developed for hand surgery, WALANT has recently gained interest in foot and ankle surgery, particularly for minor to moderate forefoot procedures such as hallux valgus correction, digital osteotomies, exostectomy, and Morton’s neuroma excision [37]. The technique involves subcutaneous and deep tissue infiltration of a diluted solution of lidocaine with epinephrine directly into the surgical field, typically 20–30 min prior to incision to allow sufficient vasoconstriction. Buffering with sodium bicarbonate is often used to reduce injection pain. The volume and pattern of infiltration are adapted according to the anatomy of the foot and the planned surgical exposure. WALANT offers several potential advantages, including elimination of tourniquet-related discomfort and decreased recovery time. It also avoids the use of proximal motor blocks, facilitating early postoperative mobilization. However, the technique has limitations. Evidence for WALANT in foot surgery is still limited to small cohort studies and case series, and high-quality randomized trials are lacking [38]. Its efficacy in procedures requiring a thigh tourniquet is questionable due to limited coverage of ischemic pain [37]. Additionally, the relatively short duration of action of lidocaine may result in suboptimal postoperative analgesia, especially if not supplemented by other systemic or regional analgesic strategies. Care must also be taken in patients with peripheral vascular disease or those at risk for local ischemia, although current literature has not shown a significant increase in complications when epinephrine is used appropriately.

## 8. Multimodal Analgesia

Effective pain management is an essential component of foot and ankle surgery, as inadequate perioperative analgesia can lead to delayed recovery, increased opioid consumption, and prolonged hospital stays. A multimodal analgesia regimen—combining different classes of analgesic medications with regional anesthesia techniques—has become the standard approach to maximize pain relief while minimizing side effects. This strategy is particularly relevant in outpatient foot surgery, where early mobilization and timely discharge are critical priorities. According to international recommendations, the multimodal analgesic regimen for hallux valgus and other common foot procedures should include a combination of paracetamol, NSAIDs or COX-2 selective inhibitors, and systemic corticosteroids [24]. These medications should be administered preoperatively or intraoperatively and continued postoperatively to maintain effective analgesia. Paracetamol remains a cornerstone of this regimen due to its favorable safety profile and risk-benefit ratio. NSAIDs and COX-2 inhibitors complement analgesia by providing anti-inflammatory effects and reducing nociceptive pain, ultimately enhancing patient comfort after surgery. Systemic corticosteroids—particularly intravenous dexamethasone—are recommended both for their antiemetic properties and for their ability to prolong the duration and effectiveness of regional anesthesia. Evidence suggests that intramuscular or oral corticosteroids offer comparable analgesic benefits, but intravenous dexamethasone is preferred because of its well-documented antiemetic effect [39]. When included in a multimodal regimen, dexamethasone can extend the duration of regional blocks and reduce the need for opioid analgesics during the immediate postoperative period [40]. Opioids should not be used routinely for pain management in foot surgery but reserved strictly as rescue medication when breakthrough pain is not adequately controlled with first-line multimodal measures [24]. Consistent evidence shows that combining regional anesthesia with multimodal pharmacological strategies significantly reduces opioid requirements without compromising pain control. By limiting routine opioid use, healthcare providers can decrease postoperative nausea, vomiting, and sedation while helping to prevent long-term opioid dependence [41].

## 9. Considerations for Out-Patient Surgery

**Figure 10:**
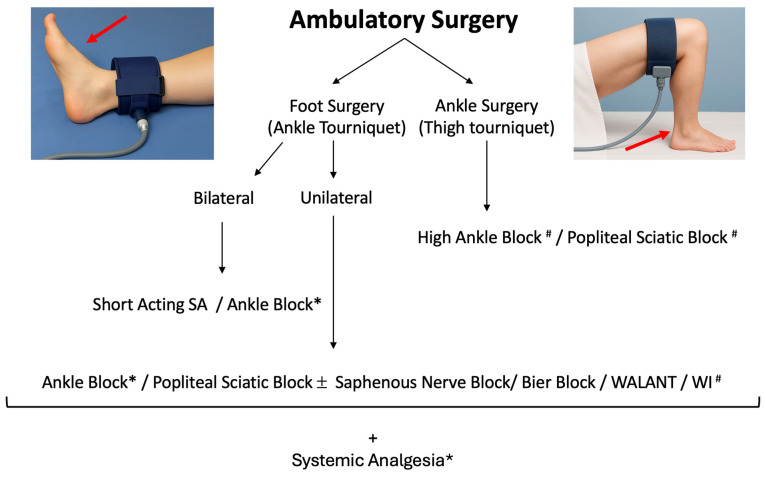
Anesthesia Techniques for Ambulatory Surgery An Overview about anesthesia and analgesia technique for foot and ankle ambulatory surgery. SA: Spinal Anesthesia; WALANT: Wide Awake Local Anesthesia No Tourniquet; WI: Wound Infiltration; * recommended; #: necessitate SA or deep sedation.

Ambulatory foot surgery has become increasingly common thanks to advances in anesthesia techniques, effective multimodal analgesia, and the spread of minimally invasive surgical procedures. Outpatient settings offer clear advantages in terms of cost savings, efficiency, and faster recovery; recent data confirm that foot and ankle surgeries can be performed safely in ambulatory surgery centers with low adverse event rates when proper patient selection and perioperative care are ensured [42]. The main challenge remains to deliver adequate analgesia without compromising surgical outcomes or delaying discharge and ambulation. Good perioperative pain management reduces postoperative complications, facilitates early mobilization, and minimizes unplanned hospital admissions for uncontrolled pain. According to the PROSPECT recommendations for hallux valgus and other common foot procedures, an ankle block is the first-choice regional technique in outpatient settings because it provides effective analgesia with minimal motor impairment, thus allowing faster recovery and discharge [24]. Wound infiltration with local anesthetic is a reasonable alternative, although it necessitates to be combined with Spinal Anesthesia or General Anesthesia. Motor-sparing regional techniques should be prioritized, especially in bilateral procedures, to avoid residual motor block that could hinder safe ambulation and increase the risk of falls at home. In this context, the use of distal blocks, such as ankle blocks, is preferable to more proximal approaches like popliteal sciatic blocks when early weight-bearing is anticipated. Sciatic nerve blocks, while effective for prolonged analgesia, are associated with a higher incidence of motor block (e.g., foot drop), which may be counterproductive in an ambulatory setting where immediate mobility is often required. When neuraxial anesthesia is indicated, the use of short-acting local anesthetics—such as prilocaine or chloroprocaine—should be favored to ensure rapid motor recovery and allow same-day discharge [43]. This is particularly important for day-case surgery to prevent delayed discharge due to prolonged motor block or urinary retention. A multimodal analgesic regimen combining paracetamol, NSAIDs or COX-2 inhibitors, and systemic corticosteroids (e.g., intravenous dexamethasone) remains essential to optimize pain control and reduce opioid consumption [24]. Opioids should be reserved strictly as rescue analgesics for breakthrough pain not controlled by first-line measures. When extended analgesia is needed, the addition of perineural adjuvants such as clonidine or dexmedetomidine may be considered to prolong block duration without increasing opioid requirements. Ultimately, careful patient selection, clear perioperative instructions, and the choice of motor-sparing regional techniques and appropriate anesthetic agents are key to achieving effective pain control while supporting early mobilization, patient safety, and timely discharge in ambulatory foot and ankle surgery. An overview of anesthesiologic options for outpatient surgery is displayed in Figure 10.

## 10. Conclusions

Effective perioperative analgesia for foot and ankle surgery relies on a tailored use of regional anesthesia techniques combined with systemic multimodal analgesic strategies. Motor-sparing blocks, ultrasound guidance, and short-acting spinal agents help balance pain control with early mobilization, which is crucial in outpatient settings. Selecting the right technique according to surgical procedure, patient needs, and safety considerations remains the key to improving outcomes, minimizing opioid use, and supporting faster recovery.

## Abbreviations

PSNB	Popliteal Sciatic Nerve Block
PNS	Peripheral Nerve Stimulation
DPN	Deep Peroneal Nerve
ATA	Anterior Tibial Artery
SPN	Superficial Peroneal Nerve
EDL	Extensor Digitorum Longus
FL	Fibularis Longus
FB	Fibularis Brevis
IVRA	Intravenous Regional Anesthesia
LAST	Local Anesthetic Systemic Toxicity
WALANT	Wide Awake Local Anesthesia No Tourniquet
